# Leishmanicidal Activity of *Piper nigrum* Bioactive Fractions is Interceded via Apoptosis *In Vitro* and Substantiated by Th1 Immunostimulatory Potential *In Vivo*

**DOI:** 10.3389/fmicb.2015.01368

**Published:** 2015-12-08

**Authors:** Garima Chouhan, Mohammad Islamuddin, Muzamil Y. Want, Hani A. Ozbak, Hassan A. Hemeg, Dinkar Sahal, Farhat Afrin

**Affiliations:** ^1^Parasite Immunology Laboratory, Department of Biotechnology, Faculty of Science, Jamia Hamdard (Hamdard University)New Delhi, India; ^2^Department of Medical Laboratories Technology, Faculty of Applied Medical Sciences, Taibah UniversityMedina, Saudi Arabia; ^3^Malaria Research Group, International Centre for Genetic Engineering and BiotechnologyNew Delhi, India

**Keywords:** Visceral leishmaniasis, antileishmanial, *Piper nigrum*, immunomodulatory, leishmanicidal, *Leishmania donovani*, apoptosis

## Abstract

Visceral leishmaniasis (VL) is a life-threatening protozoal infection chiefly impinging the rural and poor population in the tropical and sub-tropical countries. The deadly aﬄiction is rapidly expanding after its association with AIDS, swiftly defying its status of a neglected disease. Despite successful formulation of vaccine against canine leishmaniasis, no licensed vaccine is yet available for human VL, chemotherapy is in appalling state, and the development of new candidate drugs has been painfully slow. In face of lack of proper incentives, immunostimulatory plant preparations owing antileishmanial efficacy bear potential to rejuvenate awful antileishmanial chemotherapy. We have earlier reported profound leishmanicidal activity of *Piper nigrum* hexane (PNH) seeds and *P. nigrum* ethanolic (PNE) fractions derived from *P. nigrum* seeds against *Leishmania donovani* promastigotes and amastigotes. In the present study, we illustrate that the remarkable anti-promastigote activity exhibited by PNH and PNE is mediated via apoptosis as evidenced by phosphatidylserine externalization, DNA fragmentation, arrest in sub G_0_/G_1_ phase, loss of mitochondrial membrane potential and generation of reactive oxygen species. Further, *P. nigrum* bioactive fractions rendered significant protection to *L. donovani* infected BALB/c mice in comparison to piperine, a known compound present in *Piper* species. The substantial therapeutic potential of PNH and PNE was accompanied by elicitation of cell-mediated immune response. The bioactive fractions elevated the secretion of Th1 (INF-γ, TNF-α, and IL-2) cytokines and declined IL-4 and IL-10. PNH and PNE enhanced the production of IgG2a, upregulated the expression of co-stimulatory molecules CD80 and CD86, augmented splenic CD4^+^ and CD8^+^ T cell population, induced strong lymphoproliferative and DTH responses and partially stimulated NO production. PNH and PNE were devoid of any hepatic or renal toxicity. These encouraging findings merit further exploration of *P. nigrum* bioactive fractions as a source of potent and non-toxic antileishmanials.

## Introduction

Leishmaniasis comprises poverty associated neglected vector-borne diseases that are responsible for considerable morbidity and mortality all over the world. Visceral manifestation is the life-threatening form of the disease (Rogers, 2012) and is responsible for 60,000 deaths annually, causing a death toll preceded only by malaria in case of parasitic infections (Kaye and Aebischer, 2011). VL is endemic in India apart from Bangladesh, Nepal, South Sudan, Sudan, and Brazil which together account for 90% of the 200,000 to 400,000 new cases reported yearly (Chowdhury et al., 2014). Clinically cured patients often suffer from relapse in the form of post kala-azar dermal leishmaniasis and VL now poses a serious threat as common co-infection with AIDS in endemic as well as non-endemic countries (Cota et al., 2011).

The options available for VL control are limited due to non-availability of any licensed vaccine, debilitating chemotherapy which is riddled with emerging resistance, cost and toxicity, and already failing combination therapy (Garcia-Hernandez et al., 2012). Pentavalent antimonials constitute first line of drugs since their discovery more than 60 years ago; however, their use has already been abandoned in Bihar, India, due to resurgence of resistance. AmB, constitutes second line of drugs and also serves as the first choice of treatment in cases where patients become unresponsive to antimonials. However, its use is demerited by need of hospitalization, continuous monitoring of patients, prolonged duration of treatment and infusion-related side-effects (fever, chills, thrombophlebitis). More toxic but less common side-effects include hypokalaemia, nephrotoxicity, myocarditis, and even death. All these adverse toxicities coupled with high cost, limit the choice of AmB as a favorable treatment for leishmaniasis despite its tremendous efficacy. Lipid formulations of AmB although non-toxic, are expensive which is a crucial drawback as leishmaniasis-inflicted major population is chiefly rural and extremely poor. Newer drugs such as miltefosine (oral agent) and paromomycin have been registered for use in India against VL. However, miltefosine is teratogenic, causes irreversible gastro-intestinal disturbances and renal toxicity, and there are reports of drug resistance, which limits its usefulness (Sundar et al., 2006). On the other hand, parenteral route of administration and other baneful manifestations such as ototoxicity and nephrotoxicity caused by paromomycin negatively impacts its use (Sundar and Agarwal, 2011). Allopurinol is a purine analog and its antileishmanial activity was discovered about three decades ago. After Miltefosine, it was considered as another promising oral agent and was investigated in clinical trials for CL and VL but the results were not up to expectations. Despite this, it exhibited good efficacy against canine leishmaniasis and is employed in maintenance therapy for canine leishmaniasis (Monzote, 2009). Sitmaquine (8-aminoquinoline) once considered as a future oral antileishmanial drug in pipeline, is no longer in development. Sitamaquine was being developed by GlaxoSmithKline and underwent extensive phase II trials in India and East Africa where it exhibited <90% cure rates (Croft and Olliaro, 2011 and references therein).

In order to find cost-effective and efficacious alternatives, the search for new drugs is directed toward exploration of alternative sources. Plant kingdom has invariably served as an inexhaustible source for drug discovery with boundless structural and molecular diversity which has favoured the quest for more efficacious and safe therapeutics, against both parasitic and non-parasitic diseases. Use of plant extracts in addition to plant secondary metabolites for containment of antiparasitic diseases including leishmaniasis is often recommended (Wink, 2012) and it is claimed that crude or semipurified preparations of whole plant or plant parts may be more effective therapeutically (Kayser et al., 2003). World Health Organization has also promoted the use of plant extracts for oral and topical application in case of VL and CL, respectively (Fournet and Munoz, 2002). Search for plants derived antileishmanial drugs has been intensive and innumerable plants belonging to diverse families have exhibited appreciable leishmanicidal efficacy (de Carvalho and Ferreira, 2001; Sen and Chatterjee, 2011; Brito et al., 2013).

Further, as the *Leishmania* parasites incapacitate the host macrophages and impair their functioning, eventually leading to suppression of CMI (Schmid et al., 2012), an ideal approach to cure leishmaniasis should include administration of antileishmanial compounds, which can concomitantly rejuvenate the defective host immunity. Over the years, compounds with dual antiparasitic and immunomodulatory potential have gained prominence in the search for new and better leishmanicidal agents. Several plant extracts and secondary metabolites have resulted in therapeutic polarization of host immunity to treat leishmaniasis (Chouhan et al., 2014a and references therein). Thus, use of immunostimulatory plant extracts or compounds alone or in combination with existing chemotherapy can lend leishmaniasis therapy a new direction. As immunostimulatory plant fractions are composed of multiple compounds they may impact upon diverse molecular targets to eliminate the parasites thereby reducing the risk of drug resistance (Spelman et al., 2006 and references therein).

Plants of Piperaceae family used as sources of ingredients for food spices have been employed in traditional medicines since ancient times. Plant extracts from various species of genus *Piper* have demonstrated potent leishmanicidal efficacy (Chouhan et al., 2014b and references therein). *Piper nigrum* is a well-known medicinal plant exhibiting a diverse range of biological activities that have been explored in traditional medicinal systems of both new world and old world countries (Srinivasan, 2007). Immunomodulatory potential of *P. nigrum* has been well documented along with its antiprotozoal properties (Shaba et al., 2012). We have previously reported that seeds of *P. nigrum* bear profound antileishmanial activity *in vitro* and *ex vivo* (Chouhan et al., 2014b). *P. nigrum* hexane (PNH) and *P. nigrum* ethanolic (PNE) fractions of seeds inhibited the growth of *Leishmania donovani* promastigotes and amastigotes substantially in comparison to piperine (PIP), one of the bioactive compounds present in plants of *Piper* species. The plant secondary metabolites present in these fractions were also identified by GC-MS. In the present study, we extended our work to identify the mode of induced cell death by these fractions in *L. donovani* promastigotes as well as their protective efficacy *in vivo* along with evaluation of Th-1 and Th2 correlates of host immunity.

## Materials and Methods

### Materials

All the chemicals used throughout the study were procured from well-known standard companies. Sodium dihydrogen phosphate, disodium hydrogen phosphate, hydrogen peroxide, citric acid, *o*-phenylenediamine dihydrochloride, ethanol, hexane, methanol, BSA, Folin’s reagent, ammonium chloride, Trypan blue, sodium nitrite, phosphoric acid, sulphanilamide, *N*-(1-napthyl) ethylene dihyrochloride, and Tween-20 were procured from Merck. FBS was purchased from GIBCO-BRL. All other chemicals were purchased from Sigma-Aldrich unless otherwise stated.

### Methods

#### *L. donovani* Culture Maintenance

*Leishmania donovani* culture (MHOM/IN/83/AG83) was a kind gift from Dr. Nahid Ali, Indian Institute of Chemical Biology, Kolkata, West Bengal, India. *L. donovani* infection was maintained *in vivo* in BALB/c mice. *L. donovani* parasites were cultured in medium M199 plus 10% heat-inactivated FBS as described previously (Chouhan et al., 2015). Briefly, promastigotes were subcultured at regular intervals (72–96 h) after adjusting the inoculum density to 2 × 10^6^ cells ml^-1^ at 22°C.

#### Maintenance of Animals

BALB/c mice were reared in Central Animal House of Jamia Hamdard, and females, aged 6–8 weeks and weighing about 20–25 g were used for carrying out *in vivo* experiments. The study protocol was approved (Ethical approval judgment number, 499) from the JHAEC which is registered under the CPCSEA.

#### Plant Material and Extraction

*Piper nigrum* dried fruits were purchased locally, and authenticated by Dr. H.B. Singh, Taxonomist, NISCAIR, CSIR, New Delhi (voucher no. NISCAIR/RHMD/Consult/-2010-11/1440/38). The powdered plant material (100 g) was subjected to sequential extraction with hexane, ethanol and water as reported earlier (Chouhan et al., 2014b). The dried fractions were stored at –20°C until used for bioassay.

#### Determination of PS Externalization

Phosphatidylserine externalization was detected by Annexin–V FLUOS staining kit (Roche). The stationary phase promastigotes (2 × 10^6^ cells ml^-1^) were either untreated or treated with PNH, PNE, PIP, and pentamidine (500 μg ml^-1^) for 72 h at 22°C. The downstream processing was carried out according to the manufacturer’s instructions. The parasites were incubated with Annexin-V-FLOUS and PI (2 μl each) along with sample buffer (100 μl) for 15 min in dark at RT. After incubation, the samples were acquired on BD LSR-II flow cytometer, 10,000 events per sample were recorded and the dot plots of FLOUS (FL-1 channel) versus PI (FL-2 channel) were recorded and data analysis was carried out using BD FACS DIVA software (Islamuddin et al., 2014).

#### Analysis of DNA Fragmentation by TUNEL Assay

DNA fragmentation in the bioactive fractions treated (500 μg ml^-1^) and untreated parasites was detected by TUNEL assay according to the manufacturer’s instructions (Apo direct Kit, Roche). Briefly, stationary phase parasites (2 × 10^6^ ml^-1^) were treated with PNH and PNE (500 μg ml^-1^) along with appropriate controls for 72 h at 22°C. After incubation, the parasites were harvested and processed as described previously (Chouhan et al., 2015), following which the cells were incubated in buffer containing nucleotide mix (1 h in a humidified atmosphere at 37°C) provided with the kit. The promastigotes were finally washed and resuspended in PBS and acquired on BD LSR-II flow cytometer. The data was acquired (10,000 events per sample) in the form of histograms and quadrant statistics were calculated using BD FACS DIVA software.

#### Determination of Sub G_0_/G_1_ Population

The sub G_0_/G_1_ population was estimated by staining with PI according to Sarkar et al. (2008). The bioactive fractions treated (500 μg ml^-1^, 72 h, 22°C) or untreated promastigotes were harvested, fixed in 80% chilled ethanol and stored at 4°C (for at least 24 h) prior to incubation with RNase (200 μg ml^-1^, 37°C, 1 h) and further stained with PI (50 μg ml^-1^) for 20 min in dark at 22–25°C. The cells were acquired on BD LSR II flow cytometer. For each sample, 10,000 events were recorded and the cell cycle distribution was analyzed using BD FACS DIVA software.

#### Estimation of MMP, Ψm

Changes in Ψm were determined according to Chandrasekaran et al. (2013) with few modifications by staining with JC-1 (5,5′,6,6′-tetrachloro-1,1′,3,3′-tetraethylimidacarbocyanine iodide). The differential fluorescence patterns exhibited by JC-1 enable us to determine relative Ψm of a cell. In live cells, the dye accumulates inside the mitochondria forming J-aggregates which fluoresce red at 590 nm whereas, in cells undergoing apoptosis the dye is no longer retained inside mitochondria and enters cytoplasm where it fluorescence green at 530 nm. Thus, red/green ratio or fluorescence intensity at 590/530 tells us about relative Ψm of the cell. Briefly, *L. donovani* promastigotes (2 × 10^6^ ml^-1^) were treated with the bioactive fractions (500 μg ml^-1^, 72 h, 22°C) along with standard controls. Post treatment, the cells were washed twice and stained with 10 μg of JC-1 for 10 min at 37°C. The cells were again washed with PBS before acquisition in BD LSR II flow cytometer. For each sample 10,000 events were recorded, and quadrant dot plots (red, *y*-axis versus green, *x*-axis) were then generated to distinguish J-aggregates (red fluorescence) from JC-1 monomers (green-fluorescence) using BD FACS DIVA software. The ratio of J aggregates/monomers (red/green) was then plotted to estimate relative Ψm of a cell.

#### Assessment of ROS Generation

2′, 7′- dichlorodihydrofluorescein-diacetate (H_2_DCFDA) staining was performed to detect intracellular ROS generation as described previously (Chouhan et al., 2015). *L. donovani* promastigotes (2 × 10^6^ cells ml^-1^) were cultured in the presence of PNH, PNE, PIP, pentamidine (500 μg ml^-1^) or medium alone at 22°C for 72 h. Cells were then washed twice with PBS, incubated with 10 μM of H_2_DCFDA in dark at RT for 15 min, and finally acquired in BD LSR II flow cytometer, where MFI for each sample was recorded. 10,000 events for each sample were acquired, and histogram analysis of FL-1H (*x*-axis, green fluorescence) was performed. On the basis of negatively (untreated) and positively (pentamidine treated) stained cell populations, appropriate region was gated to calculate the MFI.

#### Evaluation of *L. donovani* Parasite Burden *In Vivo*

Six to eight weeks old female BALB/c mice were infected with stationary phase *L. donovani* promastigotes (2.5 × 10^7^/animal) via tail vein. Following 10 weeks, parasite burden was evaluated in three arbitrarily selected animals as described previously (Chouhan et al., 2015), after which the mice were randomly assorted into different groups (*n* = 5 per group). Infected mice were treated daily for two weeks (Eissa et al., 2011; Ferreira et al., 2014) according to dose regimen mentioned in **Table 1** (after prior *in vivo* standardization studies) except for AmB, a commercially available antileishmanial drug, taken as positive control for *in vivo* studies. AmB was administered intravenously for 5 days, alternatively at the mentioned dose (**Table 1**). Two weeks post-treatment; the mice were bled and euthanized for determining the therapeutic efficacy along with immunostimulatory potential and toxicity profile. Impression smears of liver and splenic tissue were prepared to ascertain the parasitic load in terms of Leishman-Donovan units (LDU) according to the formula (Bradley and Kirkley, 1977).

**Table 1:** Experimental design for *in vivo* studies.

Group	Regimen
Normal	Normal control/Naïve mice (Administered PBS only)
INF	Infected control (BALB/c mice infected intravenously (i.v.) with *L. donovani* promastigotes and administered PBS)
VC	0.5% dimethylsulphoxide (DMSO) in PBS
AmB	AmB administered i.v. at (5 mg/kg bw)
PNH100	PNH administered intraperitoneally (i.p.) at 100 mg/kg bw
PNH200	PNH administered i.p. at 200 mg/kg bw
PNE100	PNE administered i.p. at 100 mg/kg bw
PNE200	PNE administered i.p. at 200 mg/kg bw
PIP 200	Piperine administered i.p. at 200 mg/kg bw

$$\frac{\text{No}.\;\text{of}\;\text{amastigotes}}{\text{No}.\;\text{of}\;\text{macrophages}}\;\times \;\text{organ}\;\text{weight}\;(\text{mg})$$

Further, percent protection rendered by PNH, PNE, and other groups was calculated according to the formula:

$$\frac{{\text{LDU}}_{\text{infectedcontrol}}\;-\;{\text{LDU}}_{\text{treated}}}{{\text{LDU}}_{\text{infected}\;\text{control}}}\;\times \;100$$

#### Preparation of Leishmanial Antigens

Leishmanial freeze thawed antigen (FT) was prepared from stationary phase promastigotes (10^8^ ml^-1^) which were washed twice with PBS and subjected to six cycles of alternate freezing (–70°C, 30 min) and thawing (37°C, 15 min). Soluble leishmanial antigen (SLA) was prepared in similar manner with few additional modifications according to Ryan et al. (2002). Parasites (10^8^ ml^-1^) were subjected to eight cycles of freezing and thawing under the above mentioned conditions followed by centrifugation (5250 × *g*, 30 min). The resultant supernatant was separated and protein concentration determined by Folin and Lowry method (Lowry et al., 1951). The antigens were stored at –70°C until use.

#### Measurement of DTH

Delayed type hypersensitivity (DTH) was measured 2 weeks post-treatment in all the experimental animals. Briefly, right hind footpad of mice received FT (800 μg ml^-1^, 50 μl) whereas the left hind foot pad was injected with PBS. After 24 h of antigen inoculation, footpad swelling was measured, and the DTH response was determined as the difference in the footpad swelling between two hind foot pads according to formula (Banerjee et al., 2008):

$$\text{Foot pad swelling }(\text{mm})\;=\;{\text{Thickness}}_{\text{right food pad}}\;-\;{\text{thickness}}_{\text{left foot pad}}$$

#### Lymphoproliferation Assay by CFSE Labeling

*In vitro* recall responses were studied in lymphocytes isolated from spleen of untreated and treated animals, post 2 weeks of treatment. Single cell suspension of spleen cells was prepared as described previously (Chouhan et al., 2015), following which cells were re-suspended at 5 × 10^6^ cells ml^-1^ in serum free (incomplete) Rosewell Park Memorial Institute (RPMI)-1640 medium supplemented with 100 μg ml^-1^ streptomycin sulfate, 100 U ml^-1^ penicillin G-sodium, 0.2% sodium bicarbonate, and 25 mM HEPES. The cells were labeled with CFSE (1 μM) in dark at RT (Del-Rey et al., 2007). Following incubation, ice-cold complete RPMI-1640 medium was added to quench the reaction, the cells were washed and *in vitro* re-stimulated with SLA (12 μg ml^-1^) or ConA (5 μg ml^-1^), a non-specific mitogen that served as positive control. Normal cells without SLA stimulation were also seeded to assess the basal level of proliferation. Post 48 h, the cells were washed twice with PBS before acquisition in BD LSR II flow cytometer. BD FACS DIVA software was used for analyzing different cell populations.

#### Determination of Changes in Splenic CD4^+^ and CD8^+^ T Cell Numbers

Effect of PNH and PNE on splenic CD4^+^ and CD8^+^ T cell populations was ascertained two weeks post treatment as described elsewhere (Pathak and Khandelwal, 2009). Briefly, single cell suspension (2 × 10^6^ per tube) of splenic lymphocytes was washed twice with FACS buffer (1% BSA in PBS) and co-stained with a cocktail of monoclonal antimouse-CD4-PE and antimouse-CD8-FITC antibodies (BD biosciences, USA) at 1:200 dilution for 15 min in dark on ice. The cells were washed twice and finally acquired in BD LSR II flow cytometer. Single color controls and suitable isotype controls were also included to rule out any background fluorescence, for appropriate gating and positioning of quadrants. Respective percentages of CD4^+^ and CD8^+^ cells were determined using BD FACS DIVA software.

#### Estimation of Th1/Th2 Cytokines

The levels of INF-γ, TNF-α, IL-2 (Th1), as well as IL-4 and IL-10 (Th2) cytokines in culture supernatants of splenocytes were estimated using BD cytometry bead array kit. Post 48 h of SLA stimulation, culture supernatants from all the experimental groups were collected and processed for analysis of Th1/Th2 cytokines according to the manufacturer’s instructions using BD LSR II flow cytometer (Pourrajab et al., 2012).

#### Estimation of Serum Immunoglobulin G (IgG)-2a and IgG1 Levels

Serum levels of IgG isotypes, IgG2a and IgG1 were determined in treated or untreated mice through ELISA according to Bhowmick et al. (2014) with few modifications. Briefly, 96 well U-bottom plates were coated with leishmanial antigen FT (25 μg ml^-1^) and left overnight at 4°C. The unbound antigen was removed and plates were then blocked with blocking buffer (1% BSA in PBS), for 3–4 h at RT followed by washing (PBS + 0.05% Tween-20, 2 × 3 times) and overnight incubation with primary antibody (mice sera, 1: 1000) at 4°C. After washing, the plates were incubated with secondary antibodies, goat anti-mouse IgG2a and IgG1 (Sigma), followed by washing and incubation with tertiary antibody, i.e., rabbit anti-goat IgG conjugated with horseradish peroxidase (1: 10,000). The substrate solution (*o*-phenylenediamine dihydrochloride, 0.8 mg ml^-1^ in phosphate citrate buffer, pH 5.0, containing 0.04% H_2_O_2_) was added after appropriate washing. After 10 min of incubation, the absorbance measured at 450 nm in an ELISA plate reader.

#### Analysis of Changes in Expression of CD80/CD86 Co-stimulatory Molecules

Phenotyping of co-stimulatory molecules (CD80 and CD86) was performed by surface staining of murine peritoneal macrophages from treated or untreated mice as described previously (Chouhan et al., 2015). 2 × 10^6^ macrophages per sample were washed twice with FACS buffer and co-stained with APC conjugated antimouse-CD80, antimouse-CD86-PeCy7 (1: 200 dilution) for 15 min in dark at RT. Suitable controls including single color and isotype controls were accordingly prepared to negate any background fluorescence, adjustment of compensation and to assess staining intensity. A total of 50,000 events were acquired and percentages of cells positive for CD80 and/or CD86 were calculated after proper gating and analysis.

#### Estimation of NO Production in BALB/c Mice Splenocytes

Nitric oxide was estimated in culture supernatants of SLA stimulated or unstimulated splenocytes after 48 h of culture using Griess reagent as described elsewhere (Roychoudhury et al., 2011). Sodium nitrite was used to generate a standard curve and nitrite concentration was calculated using specific OD values at 540 nm, generated in triplicates.

#### Assessment of Hepatic and Renal Toxicity

The toxicity profile was tested in infected as well as treated mice (five per group). The mice were administered bioactive fractions, PIP, and AmB at previously indicated doses (**Table 1**) for 2 weeks. Two weeks post-treatment, the mice were bled and sera were separated and stored at –70°C. Serum levels of liver enzymes such as SGPT, SGOT, ALP, and serum concentrations of urea and creatinine to ascertain renal toxicity were estimated using commercially available kits (Span Diagnostics Ltd., Surat, India) according to the manufacturer’s instructions (Islamuddin et al., 2014).

### Flow Cytometry Analysis

All the flow cytometry data was acquired on BD LSR II flow cytometer. For all *in vitro* and *in vivo* experiments 10,000 and 50,000 events were acquired, respectively. Cell debris, characterized by a low side scattered light (SSC)/forward scattered light (FSC) was excluded from analysis. All the analyses were carried out using BD FACS DIVA software.

### Statistical Analysis

All *in vitro* experiments were performed at least thrice and the results represented are from one of the three independent experiments and expressed as mean ± SEM of the samples in triplicate. The *in vivo* study was carried out twice with five mice per group, and the results shown are from one of the two independent experiments and expressed as mean ± SEM. Statistical analysis was performed using Graph-Pad Prism 5 software and the statistical significance was calculated by ANOVA followed by Tukey’s multiple comparison test. Differences were considered statistically significant at *P* < 0.05. Statistical significance is indicated with the help of appropriate symbols in graphs or explained in respective result section.

## Results

### Detection of Apoptosis in *L. donovani* Promastigotes by Bioactive Fractions

#### Visualization of PS Externalization

**FIGURE 1:**
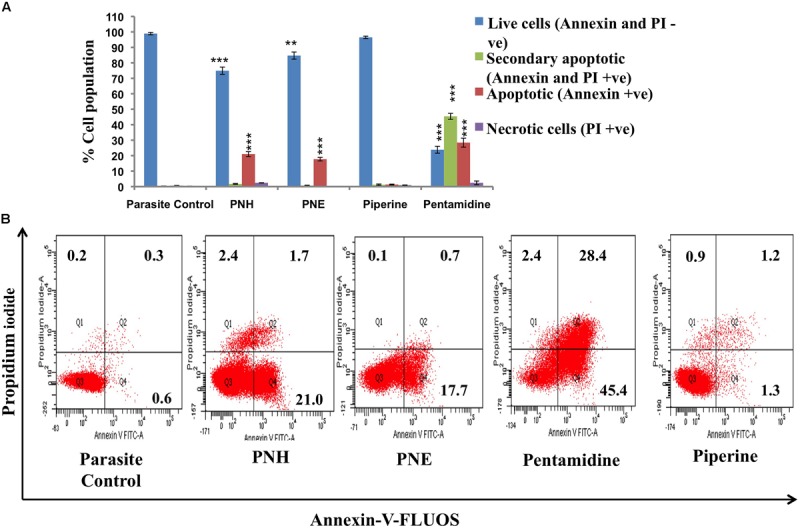
**Visualization of PS exposure. (A)** Promastigotes (2 × 10^6^ ml^-1^) after treatment with test fractions and compounds (500 μg ml^-1^) for 72 h at 22°C were co-stained with Annexin-V-FLUOS and PI. The bar graph depicts percentages of live, apoptotic, secondary apoptotic and necrotic cells for different test samples. ^∗∗∗^*P* < 0.001 and ^∗∗^*P* < 0.01 with respect to parasite control. **(B)** FL-1 (*x*-axis) vs. FL-2 (*y*-axis) dot plots depicting live (both annexin and PI negative, lower left quadrant), necrotic (PI positive, upper left quadrant), apoptotic (annexin positive, lower right quadrant) and secondary necrotic (both annexin and PI postive) *Leishmania* parasites.

PS externalization as a characteristic attribute of apoptosis was examined in PNH and PNE treated samples. Percentage of apoptotic, necrotic, late, or secondary apoptotic and live cells were deduced from quadrant statistics. 21.0% and 17.7% of treated promastigotes became Annexin positive in case of PNH and PNE, respectively (**Figures 1A,B**). A small proportion of late apoptotic cells (both Annexin and PI positive, 1.7% in PNH, 0.7% in PNE) along with apoptotic cells (PI positive, 2.4% in PNH, 0.1% in PNE) were also observed, which indicated compromised membrane integrity. In case of pentamidine, 45.4% cells were observed to be apoptotic whereas 28.4% cells were found to be secondary apoptotic. 2.5% cells underwent apoptosis following treatment with PIP whereas apoptotic toll in control parasites was 0.6%. Thus our data indicates that the mode of induced cell death by the bioactive fractions was primarily apoptosis.

#### Assessment of DNA Fragmentation

**FIGURE 2:**
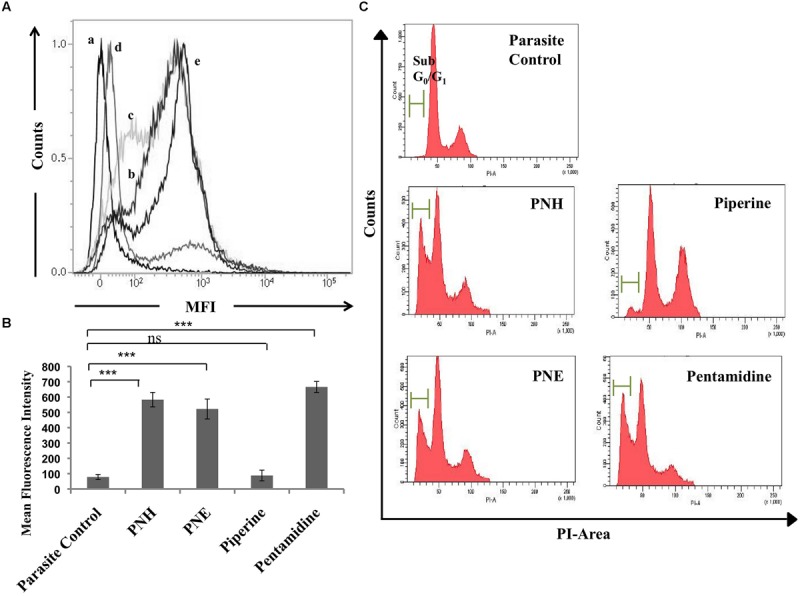
**Study of apoptotic correlates post-treatment with bioactive fractions. (A)** DNA fragmentation in treated promastigotes illustrated by TUNEL assay. The promastigotes (2 × 10^6^ ml^-1^) were treated with bioactive fractions and compounds (500 μg ml^-1^) for 72 h at 22°C after which the cells were processed according to manufacturer’s instructions and acquired on BD LSR II flowcytometer. Histograms depict shift in MFI of (b) PNH, (c) PNE, (d) piperine, and (e) pentamidine with respect to (a) parasite control. **(B)** Bar graph representing the specific MFI values indicative of TUNEL positivity in PNH, PNE and pentamidine treated samples. ^∗∗∗^*P* < 0.001 and ns = non-significant with respect to parasite control. **(C)** Detection of sub G_0_-G_1_ or apoptotic population. Promastigotes (2 × 10^6^ ml^-1^) were untreated or treated (500 μg ml^-1^, 72 h, 22°C) with bioactive fractions and processed for cell cycle analysis as described in methods.

Apoptosis is characterized by rapid DNA fragmentation, which can be quantified flow cytometrically by TUNEL assay. Treatment with PNH and PNE resulted in nuclear DNA fragmentation as evidenced by an increase in dUTP-FLOUS labeling (**Figures 2A,B**). In PNH and PNE treated samples, MFI increased to 583 (*P* < 0.001) and 522 (*P* < 0.001), respectively, in comparison with parasite control (MFI = 78), respectively. PIP treated promastigotes exhibited a non-significant increase in MFI (MFI = 88, *P* > 0.05) whereas pentamidine treated promastigotes underwent extensive DNA fragmentation (MFI = 666, *P* < 0.001).

#### Cell Cycle Analysis

Cell cycle analysis unveils distribution of cells in different interphase stages based on their DNA content. The cells with fragmented DNA as a result of any treatment are detected in sub G_0_–G_1_ phase, which is indicative of apoptosis. In PNH and PNE treated samples, 30.1% (*P* < 0.001) and 23.7% (*P* < 0.001) cells underwent apoptosis whereas in PIP treated samples, only 3.6% ± 0.4 cells were found to be apoptotic (*P* > 0.05). In pentamidine treated samples 28.2% ± 1.15 (*P* < 0.001) turned apoptotic whereas in parasite control only 0.4% ± 0.55 cells were detected in sub G_0_–G_1_ phase (**Figure 2C**).

#### Determination of Changes in Ψm

**FIGURE 3:**
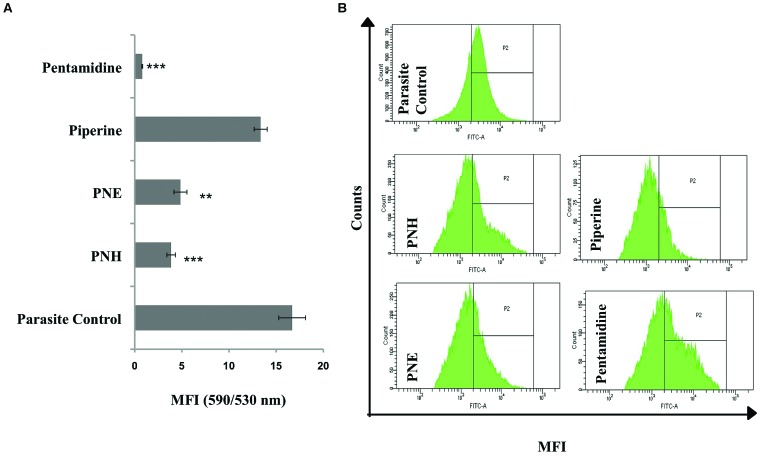
**Effect of *Piper nigrum* bioactive fractions on intracellular events leading to apoptosis. (A)** Study of mitochondrial membrane depolarization. *Leishmania donovani* promastigotes (2 × 10^6^ ml^-1^) were incubated with different bioactive fractions or test compounds (500 μg ml^-1^, 72 h, 22°C) and stained with JC-1 as described in methods. ^∗∗∗^*P* < 0.001 and ^∗∗^*P* < 0.01 in relation to parasite control. **(B)** ROS generation upon bioactive fractions treatment. ROS generation was monitored in *L. donovani* promastigotes (2 × 10^6^ ml^-1^) post 72 h of treatment (500 μg ml^-1^) with different bioactive fractions. Following acquisition of H_2_DCFDA stained parasites, the histograms were plotted where P2 gated region depicts shift in MFI. The shift in MFI was significant in case of PNH, PNE, and pentamidine (*P* < 0.001) and non-significant in case of piperine (*P* > 0.05) as indicated in results.

Since *Leishmania* parasites possess a single mitochondrion, the loss of Ψm and subsequent activation of apoptosis cascade becomes detrimental to the survival of organism. The changes in Ψm can be well depicted by staining with JC-1 whose differential fluorescence patterns inside (red) and outside (green) of mitochondria represent changes in Ψm. Typically, Ψm is represented as ratio of fluorescence at 590/530 nm or red/green fluorescence where a decrease in the ratio indicates loss of Ψm. In our study, PNH and PNE induced mitochondrial membrane depolarization (**Figure 3A**) as evidenced by decrease in 590/530 nm ratio in PNH (3.86 ± 0.45), PNE (4.85 ± 0.69), and pentamidine treated samples (0.84 ± 0.08) whereas PIP treatment induced non-significant changes in Ψm (13.35 ± 0.69) with respect to parasite control.

#### Estimation of ROS Generation

ROS, an important mediator in induction of apoptosis was probed by staining with H_2_DCFDA, which upon cleavage with free radicals generates green fluorescence. In control promastigotes without any treatment, basal level of ROS was detected (MFI = 3381 ± 50) whereas a sharp shift in MFI (*P* < 0.001) of PNH and PNE treated samples corresponding to 9027 ± 250.5 and 8072 ± 394, respectively (**Figure 3B**) indicated generation of ROS. PIP induced an insignificant shift in green fluorescence (MFI = 3735 ± 130, *P* > 0.05) whereas in pentamidine treated promastigotes, enhanced ROS production was well apparent (MFI = 10037 ± 401.5, *P* < 0.001).

### *In Vivo* Antileishmanial and Immunomodulatory Potential of Bioactive Fractions

#### Reduction in Parasite Burden and Hepatosplenomegaly

**FIGURE 4:**
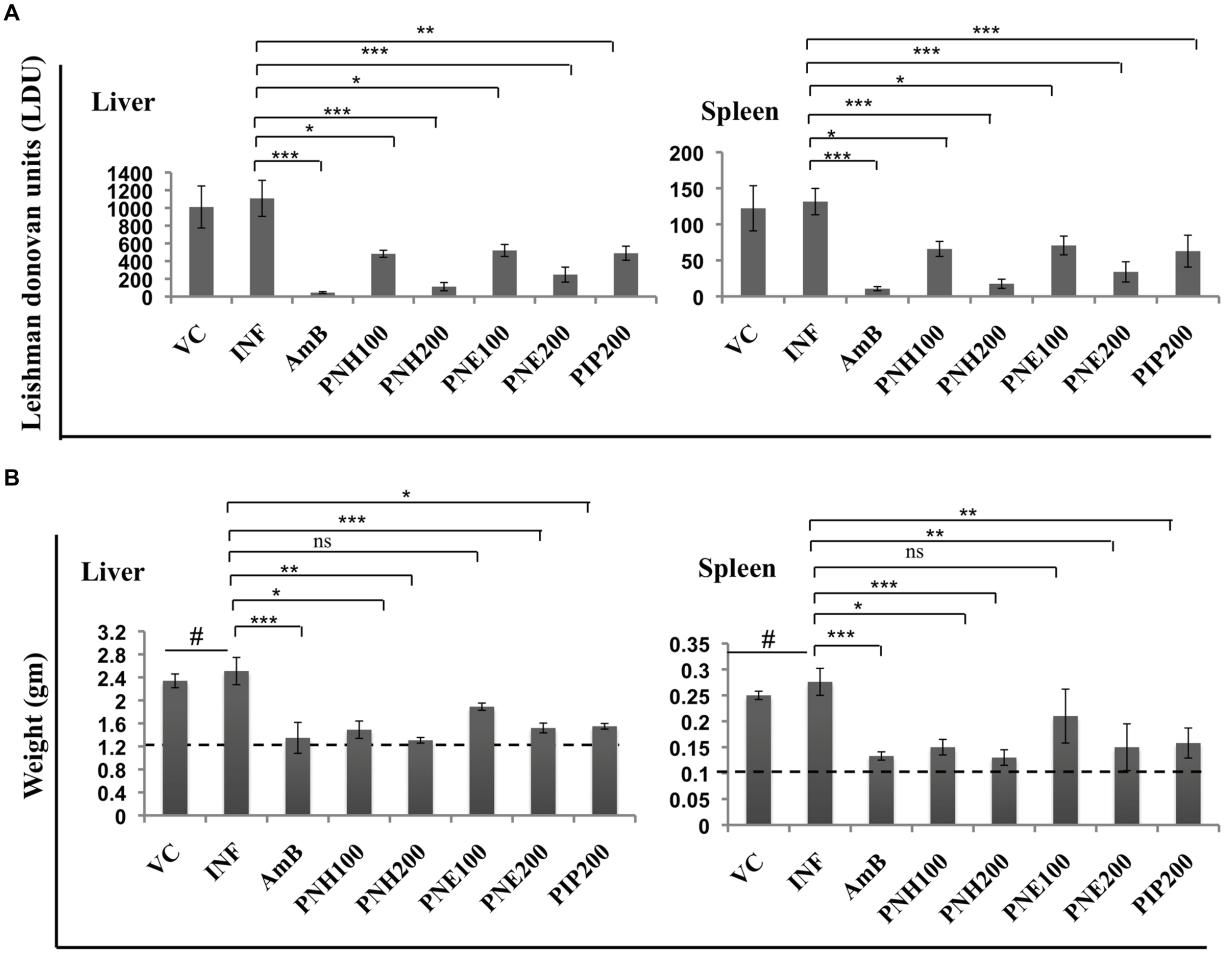
***In vivo* therapeutic potential of *P. nigrum* bioactive fractions. (A)** Estimation of parasite burden in untreated and bioactive fractions treated mice. VC = vehicle control (0.5% DMSO in PBS), INF = Infection control, AmB = AmB at 5 mg/kg bw, PNH100 = *P. nigrum* hexane fraction at 100 mg/kg bw, PNH200 = *P. nigrum* hexane fraction at 200 mg/kg bw, PNE100 = *P. nigrum* ethanolic fraction at 100 mg/kg bw, PNE200 = *P. nigrum* ethanolic fraction at 200 mg/kg bw and PIP200 = Piperine at 200 mg/kg bw. *L. donovani* infected BALB/c mice were either untreated or subjected to different treatments followed by estimation of hepatic and splenic parasite burden as described in methods. ^∗∗∗^*P* < 0.001, ^∗∗^*P* < 0.01, and ^∗^*P* < 0.05 in relation to INF group. **(B)** Differences in liver and spleen weights amongst various experimental groups. The dotted line represents average weight of respective organs in normal mice. ^#^*P* < 0.001 in comparison to naive mice, for rest ^∗∗∗^*P* < 0.001, ^∗∗^*P* < 0.01, ^∗^*P* < 0.05, and ns = non-significant with respect to INF.

*In vivo* antileishmanial efficacy of *P. nigrum* bioactive fractions was assessed in *L. donovani* infected BALB/c mice. Infected control and vehicle treated animals displayed heavy parasite burden. LDU equated to 1107.95 and 131.50 in liver and spleen, respectively, in INF group whereas in VC group, LDU corresponded to 1010.53 (liver) and 122.24 (spleen). PNH (200 mg/kg bw) caused substantial reduction in parasite load in both liver (LDU = 111.80, 89.9% protection, *P* < 0.001) and spleen (LDU = 17.52, 86.66% protection, *P* < 0.001). PNE (200 mg/kg bw) also considerably reduced the parasite burden (*P* < 0.001), in liver (LDU = 247.52, 87.76% protection), and spleen (LDU = 34.02, 85.55% protection). At lower doses, PNH at 100 mg/kg bw partially but significantly reduced the LDU to 482.48 (*P* < 0.05) and 65.85 (*P* < 0.05) in liver and spleen, respectively, with percent protection corresponding to 56.44%, and 49.93% for liver and spleen, respectively. 100 mg/kg bw of PNE treatment was the least effective in reducing parasite burden with LDU = 520.26 (liver, *P* < 0.05), and 70.65 (spleen, *P* < 0.05) conferring percent protection equivalent to 39.65 and 35.93% in liver and spleen, respectively. At 200 mg/kg bw, PIP also exhibited partial protection (*P* < 0.05), with protection levels of 55.86 and 49.93% for liver and spleen, respectively. For, AmB treatment percent protection corresponded to 95.97% (liver, *P* < 0.001) and 91.84% (spleen, *P* < 0.001), respectively, (**Figure 4A**).

The notable reduction in parasite burden following bioactive fractions was also accompanied by restoration of liver and spleen weights to normal range. INF and VC group animals exhibited significant elevation (*P* < 0.001) in liver and spleen weight in comparison to uninfected mice, which were successfully restored to normal levels by AmB as well as by PNH (200 mg/kg bw) treatment. PNE (200 mg/kg bw) treatment also declined the hepatic (*P* < 0.001) and splenic weights (*P* < 0.01), followed closely by PIP treatment, which also demonstrated similar effect (*P* < 0.05), whereas changes after PNE 100 mg/kg bw treatment were less apparent (*P* > 0.05) (**Figure 4B**).

#### DTH Response Post Treatment with PNH and PNE

**FIGURE 5:**
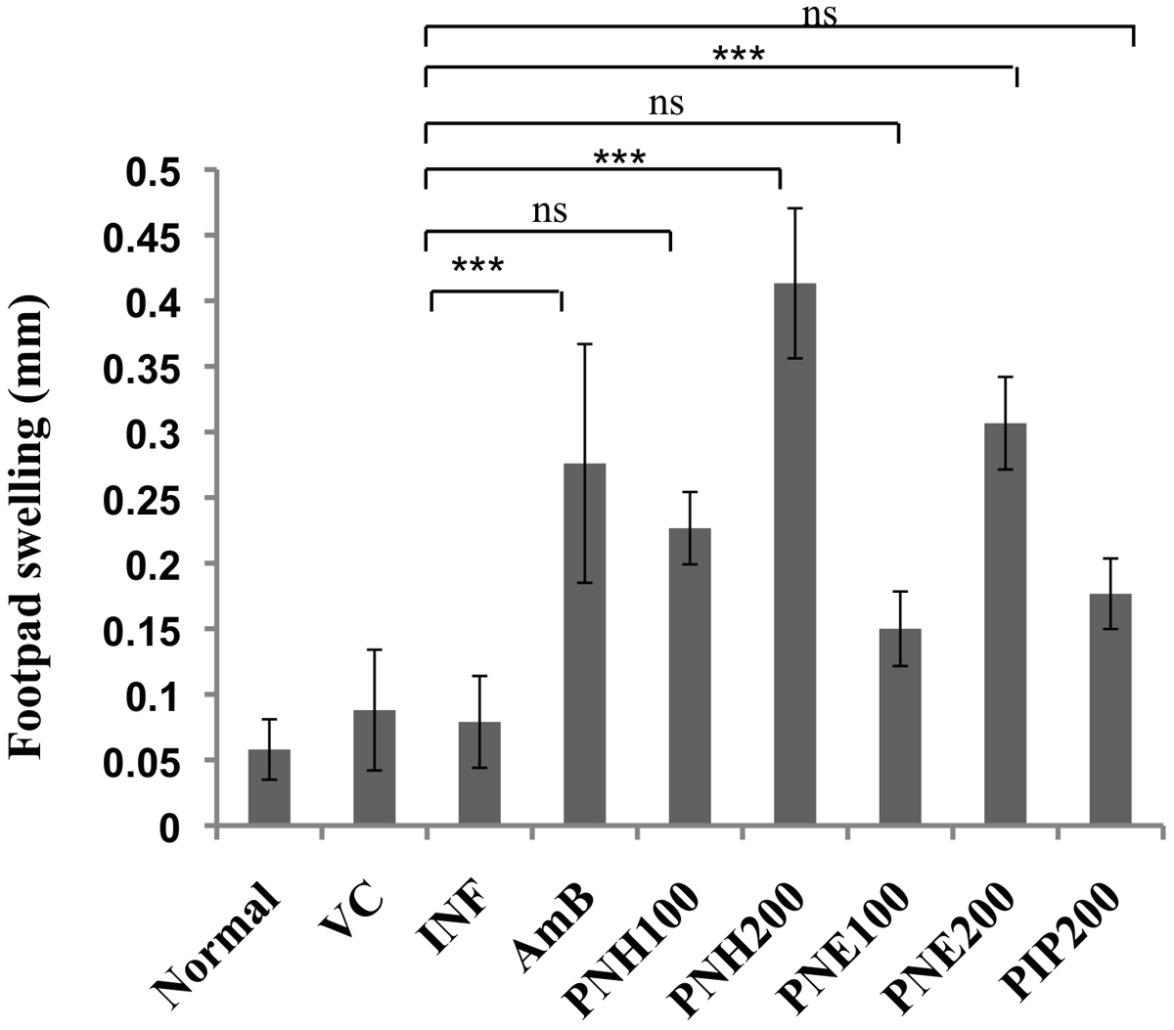
**Evaluation of DTH response.** After two weeks of treatment, animals were either inoculated with FT or PBS alone in hind footpads and DTH was measured post 24 h as described in methodology section. ^∗∗∗^*P* < 0.001 and ns = non-significant in comparison to INF.

A positive DTH reaction symbolizes successful recovery from VL without relapse in infection and activation of CMI. Thus, DTH was evaluated in *L. donovani* infected BALB/c mice post treatment with bioactive fractions, after 2 weeks interval. PNH (200 mg/kg bw) treated animals exhibited the highest DTH response which was ∼5.2-fold (*P* < 0.001) higher than infected mice. PNE treatment at 200 mg/kg bw also resulted in significant enhancement in DTH response of ∼3.49-fold (*P* < 0.001). Induction in DTH response was less potent and partial at 100 mg/kg bw dose of PNH, PNE and PIP (*P* > 0.05) whereas AmB treatment resulted in substantial elevation (∼3.49-fold, *P* < 0.001) in DTH response (**Figure 5**).

#### Effect of Bioactive Fractions on Proliferative Capacity of Splenocytes after *In Vitro* Restimulation with SLA

**FIGURE 6:**
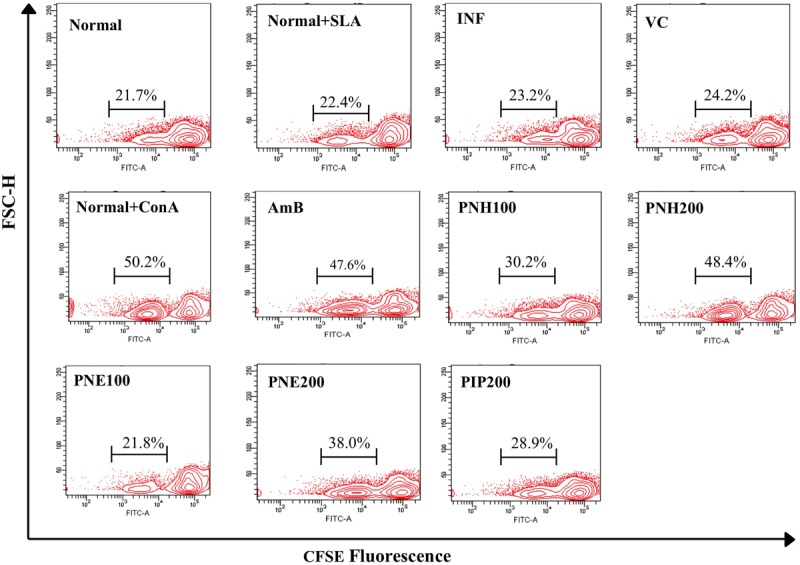
**Assessment of lymphoproliferative responses in *L. donovani* infected BALB/c mice.** Post 2 weeks treatment, RBCs free single cell suspension of splenocytes was labeled with CFSE and *in vitro* restimulated with SLA. The contour plots depict CFSE fluorescence where a shift in MFI toward left denotes loss of CFSE labeling and in turn, lymphoproliferation. Percentage of CFSE positive cells in respective samples in the presence or absence of SLA stimulation is indicated.

During progression of VL, T cells become inert to leishmanial antigens with subsequent suppression of their proliferative capacity and successful treatments are often capable of restoring the defective T cell responses. To assess the magnitude of lymphoproliferation post PNH and PNE treatments, splenocytes were labeled with CFSE. In general, CFSE fluorescence becomes half of its original after each cell division, thus a shift in CFSE fluorescence toward left of the x-axis indicates cell proliferation. Marginal numbers of CFSE positive population was observed in normal cells with or without SLA stimulation (**Figure 6**) indicating basal level of proliferation. Post-SLA stimulation, strong lymphoproliferative responses were evident in PNH and PNE treated groups. Significantly enhanced levels of CFSE positive populations were detected in PNH (48.4% ± 5.39, *P* < 0.001) and PNE (44.8% ± 2.97, *P* < 0.01) treated groups, respectively, at 200 mg/kg bw in comparison to INF (23.2% ± 3.52). At 100 mg/kg bw, lymphoproliferative responses were only partially stimulated (*P* > 0.05) with 30.2% ± 6.09, and 21.8% ± 4.12 CFSE positive cells observed in PNH and PNE treated samples, respectively. Only 28.9% cells underwent proliferation with PIP treatment and the extent of response was insignificant in comparison to INF group (*P* > 0.05). AmB specifically enhanced *in vitro* recall response after SLA stimulation (47.6% ± 2.64). ConA stimulation also resulted in significant level of proliferation with percentage of CFSE positive cells leaping up to 50.2% ± 1.47.

#### Analysis of Changes in Splenic CD4^+^ and CD8^+^ T Populations

**FIGURE 7:**
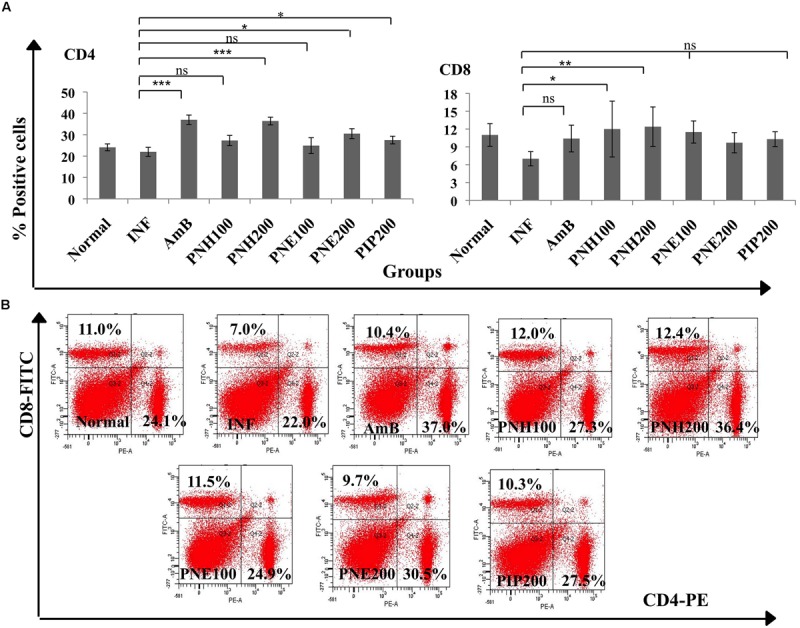
**Determination of changes in splenic T cell populations. (A)** To distinguish CD4^+^ and CD8^+^ T cell population, co-staining with anti-CD4-PE and anti-CD8-FITC antibodies was performed as described in the Section “Materials and Methods”. Bar graphs display significant and non-significant changes in CD4^+^ and CD8^+^ T cell numbers post-treatment with bioactive fractions. ^∗∗∗^*P* < 0.001, ^∗∗^*P* < 0.01, ^∗^*P* < 0.05, and ns = non-significant with respect to INF. **(B)** Dot plots also depicting the changes in splenic CD4^+^ and CD8^+^ T cell numbers after treatment with *P. nigrum* bioactive fractions.

Both CD4^+^ and CD8^+^ T cell populations are known to induce parasite clearance by initiating Th1 biased immune response. The phenotypic analysis of CD4^+^ and CD8^+^ expression revealed that *P. nigrum* bioactive fractions enhanced T cell subpopulations (**Figures 7A,B**). Expression of both CD4^+^ and CD8^+^ T cells was downregulated in INF group to 22.0% ± 2.1 (CD4^+^), and 7% ± 1.2 (CD8^+^) in comparison to naïve mice (CD4^+^= 24.1% ± 1.6, CD8^+^= 11% ± 1.9). CD4^+^ T cell population was elevated to 36.4% ± 1.80 (*P* < 0.001) and 30.5% ± 2.3 (*P* < 0.05), respectively, in case of PNH and PNE at 200 mg/kg bw treatment. At 100 mg/kg bw of PNH and PNE, the changes were less pronounced and CD4^+^ population was restored to normal values of 27.3% ± 2.4 and 24.9% ± 3.7 (*P* > 0.05), respectively. PIP treatment resulted in modulation of CD4^+^ expression in normal range (27.5% ± 1.8, *P* > 0.05) whereas AmB treatment significantly upregulated the number of CD4^+^ positive cells to 37.0% ± 2.2 (*P* < 0.001).

PNH at both the doses (100 and 200 mg/kg bw) restored the CD8^+^ T cell numbers to normal range with 12% ± 4.7 (*P* < 0.05) and 12.4% ± 3.32, (*P* < 0.01), respectively. PNE at 100 and 200 mg/kg bw resulted in insignificant immunomodulation with 11.5% ± 1.86 and 9.7% ± 0.8 (*P* > 0.05) CD8^+^ expression, respectively. PIP and AmB treatment resulted in restoration of CD8^+^ population to normalcy, 10.3% ± 1.25 and 10.4% ± 2.16 (*P* > 0.05) respectively.

#### Determination of Changes in Th1/Th2 Cytokine Levels

**FIGURE 8:**
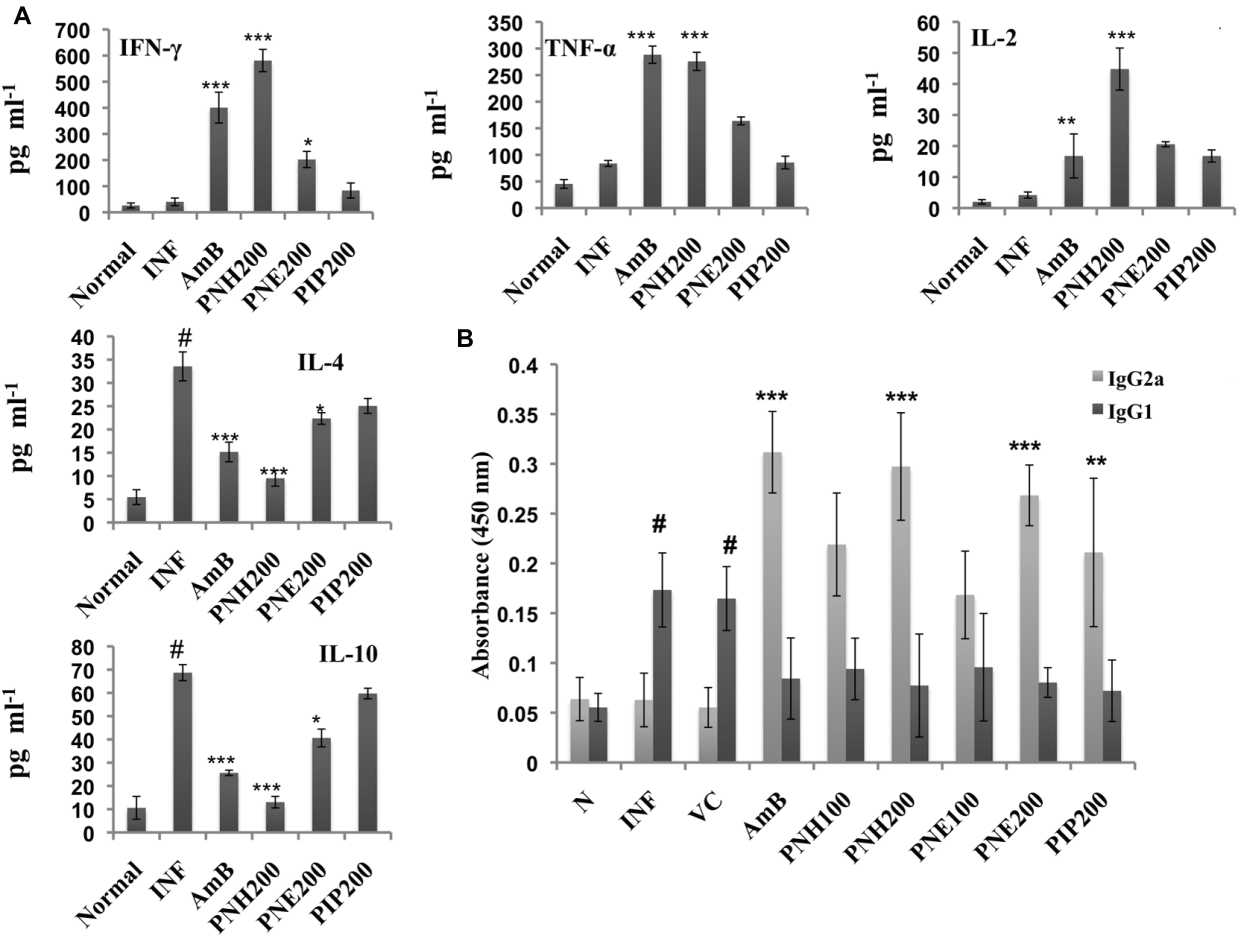
**Analysis of Th1 subset potentiation. (A)** Estimation of Th1 and Th2 cytokine levels. INF-γ, TNF-α, IL-2, IL-4, and IL-10 were quantified in culture supernatants of splenocytes post 48 h of SLA stimulation as described in the Section “Materials and Methods”. ^#^*P* < 0.001 in comparison to normal group, ^∗∗∗^*P* < 0.001, ^∗∗^*P* < 0.01, and ^∗^*P* < 0.05 with respect to INF. **(B)** IgG isotyping in sera of untreated and differently treated BALB/c mice. Leishmanial antigen specific IgG2a and IgG1 isotypes were quantified in BALB/c mice serum samples by ELISA. #,indicates significant elevation in IgG1 levels in INF and VC group in comparison to naïve mice. ^∗∗∗^*P* < 0.001, and ^∗∗^*P* < 0.01 with respect to INF.

The outcome of VL is largely dependent upon the cytokine milieu. Th2 cytokines dominate the diseased state whereas Th1 cytokines predominantly characterize the recovery state. Th1/Th2 cytokine levels were measured in the culture supernatants of splenocytes stimulated with SLA. PNH at 200 mg/kg bw induced significant levels (*P* < 0.001) of INF-γ, TNF-α and IL-2 production and waned IL-4 and IL-10 levels (*P* < 0.001) in comparison to INF (**Figure 8A**). PNE (200 mg/kg bw) partially elevated INF-γ (*P* < 0.05) and simultaneously declined IL-4 (*P* < 0.05) levels. However, there was insignificant increase in TNF-α (*P* > 0.05) and IL-2 (*P* > 0.05) and less conspicuous decrease in IL-10 (*P* > 0.05) levels by PNE in comparison to INF. PIP (200 mg/kg bw) failed to stimulate INF-γ, TNF-α and IL-2 production significantly (*P* > 0.05) and also marginally affected IL-4 and IL-10 production (*P* > 0.05). AmB treatment successfully enhanced the production of Th1 cytokines and curtailed Th2 cytokines production (*P* < 0.001).

#### Enhancement in Serum IgG2a Levels Post Treatment with *P. nigrum* Bioactive Fractions

IgG2a and IgG1 serve as surrogate markers for Th1 and Th2 activation since their production is stimulated by INF-γ and IL-4, respectively. At 200 mg/kg bw, PNH and PNE treatment stimulated ∼4.72-fold (*P* < 0.001) and ∼4.26-(*P* < 0.01) folds more IgG2a production with respect to untreated animals (**Figure 8B**). PNH and PNE at 100 mg/kg bw induced ∼3.48- and ∼2.67-folds increase in IgG2a production (*P* > 0.05). IgG2a levels were enhanced by ∼3.45-fold (*P* > 0.05) after PIP treatment whereas sera from AmB treated mice displayed maximum induction of IgG2a (∼4.95-fold). IgG1 levels were significantly increased in INF and VC groups (*P* < 0.001) with respect to naive mice. IgG1 levels were substantially lowered in all other treatment groups with lesser variations amongst the two doses (*P* < 0.05).

#### Analysis of Changes in CD80/CD86 Expression Profile

**FIGURE 9:**
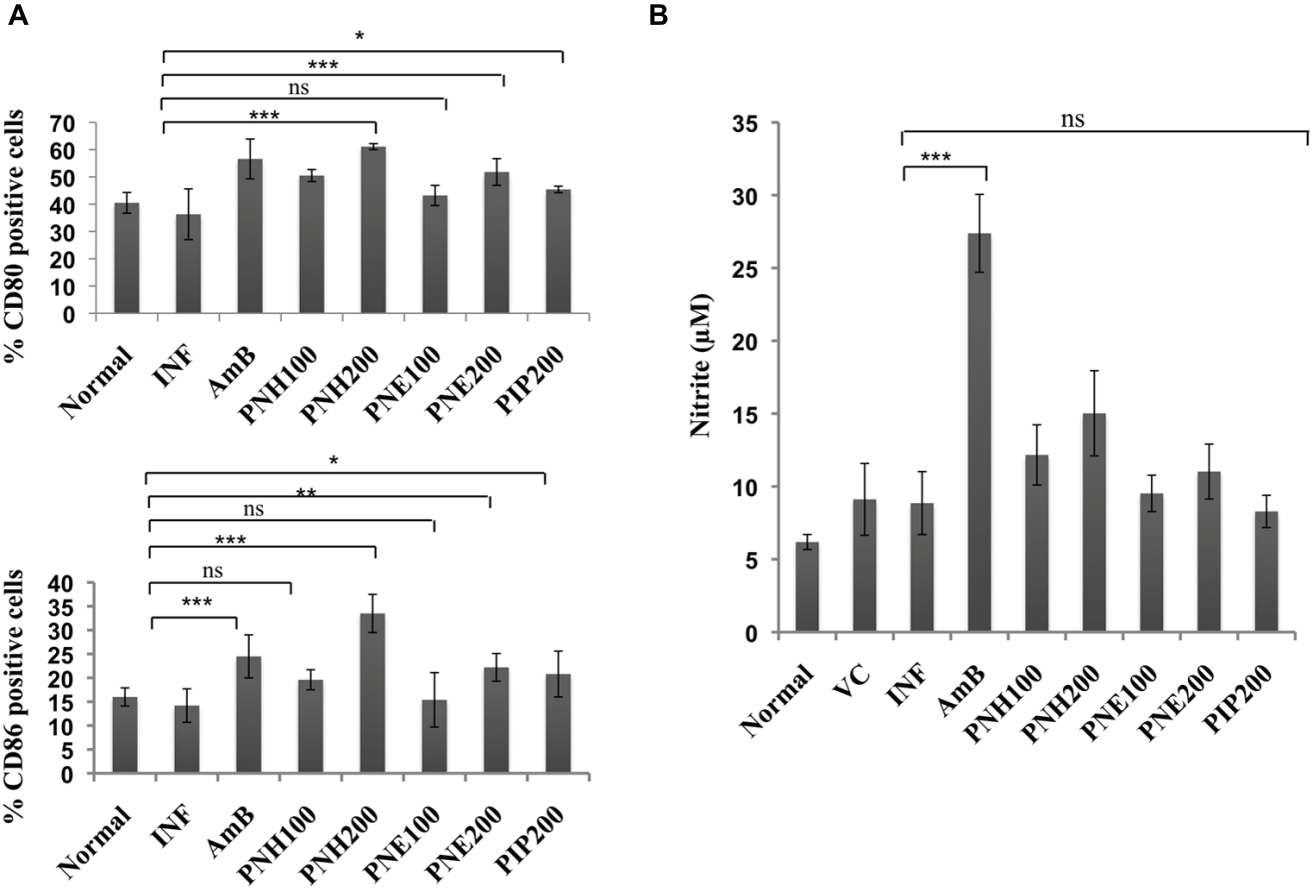
**Assessment of immunomodulatory effect of bioactive fractions on murine macrophages. (A)** Estimation of CD80 and CD86 expression. Peritoneal macrophages from different treatment groups were exposed to surface staining with antimouse-CD80-APC and antimouse-CD86-PeCy7 along with appropriate controls. Individual percentages of CD80 and CD86 positive cells for each group were deduced with the help of BD FACS DIVA software. ^∗∗∗^*P* < 0.001, ^∗∗^*P* < 0.01, ^∗^*P* < 0.05, and ns = non-significant with respect to INF. **(B)** Assessment of *in vivo* NO production. Culture supernatants from splenocytes of all the experimental groups were isolated and incubated with equal volume of griess reagent for NO estimation as described in methodology section. ^∗∗∗^*P* < 0.001 and ns = non-significant with respect to INF.

Co-stimulatory molecules strengthen the T cell-major histocompatibility complex interaction for proper activation of T cells thereby substantiating the effector immune response. Surface phenotyping for CD80 and CD86 expression was thus performed and changes in CD80 and CD86 expression were analyzed. In naïve mice, CD80 and CD86 expression corresponded to 40.5% and 16.0%, respectively (**Figure 9A**). CD80 expression was enhanced after treatment (200 mg/kg bw) with PNH (61.1%, *P* < 0.001), and PNE (51.8%, *P* < 0.001) with respect to INF (36.3%). At 100 mg/kg bw, the CD80 expression was enhanced to 50.5% (PNH, *P* < 0.001), and 43.2% after PNE (*P* > 0.05) treatment. AmB treatment also elevated the CD80 expression to 56.6% (*P* < 0.001) whereas in case of PIP treatment elevation was partial (45.4%, *P* < 0.05) but significant with respect to INF. At 200 mg/kg bw, CD86 expression was enhanced up to 33.5% (*P* < 0.001) and 22.2% (*P* < 0.01) by PNH and PNE, respectively, in comparison to INF (14.2%). The bioactive fractions at 100 mg/kg bw demonstrated non-significant (*P* > 0.05) partial modulation of CD86 expression. CD86 expression was stimulated up to 19.6 and 15.4% in case of PNH and PNE, respectively. PIP resulted in partial and significant modulation of CD86 expression to 20.8% (*P* < 0.05) and AmB treatment particularly enhanced CD86 expression up to 24.5% (*P* < 0.01).

#### Determination of NO Production

Nitric oxide generation is impaired during *Leishmania* infection, which is critical for termination of the parasite growth. All the treatment groups only marginally (*P* > 0.05) modulated NO levels except for AmB, which significantly upregulated NO production (*P* < 0.001). PIP treatment also failed to generate any significant changes in NO levels (*P* > 0.05) (**Figure 9B**).

#### Toxicity Profile of *P. nigrum* Bioactive Fractions *In Vivo*

*In vivo* adverse toxicity of bioactive fractions was assessed at 200 mg/kg bw in *L. donovani* infected mice. The serum levels of SGOT (*P* < 0.001), SGPT (*P* < 0.01), and ALP (*P* < 0.001) were particularly enhanced in INF and VC groups with respect to untreated mice. In all the treatment groups, the enzyme levels were found in normal range (*P* < 0.01) indicating no adverse effect of the bioactive fractions on hepatic function (**Table 2**). Renal toxicity was evaluated in terms of serum levels of creatinine and urea. Colorimetric estimation revealed that concentration of the metabolites was significantly enhanced in INF and VC group, which was restored to normal range after treatment with PNH and PNE (*P* < 0.001). None of the other groups exhibited significant renal toxicity (*P* < 0.001). No adverse effects were observed after AmB treatment.

**Table 2:** Estimation of serum levels of liver enzymes and kidney metabolites.

Groups	SGOT (U/L)	SGPT (U/L)	ALP (KA)	Creatinine (mg/dl)	Urea (mg/dl)
Normal	24.09 ± 3.64	33.69 ± 6.33	8.49 ± 0.43	1.05 ± 0.12	26.19 ± 1.87
INF	69.00 ± 4.49	81.67 ± 4.5	12.99 ± 0.98	3.41 ± 0.36	63.15 ± 0.57
VC	66.32 ± 5.46	80.16 ± 2.06	11.32 ± 0.89	2.99 ± 0.57	67.89 ± 4.56
AmB	27.72 ± 4.78	59.51 ± 6.30	4.15 ± 0.50	1.53 ± 0.36	33.54 ± 6.92
PNH200	23.18 ± 5.01	46.54 ± 7.33	5.96 ± 0.67	1.36 ± 0.18	34.65 ± 9.96
PNE200	31.50 ± 7.56	53.45 ± 5.66	5.23 ± 0.99	1.34 ± 0.22	38.33 ± 8.56
PIP200	26.95 ± 3.11	35.83 ± 3.90	5.61 ± 0.67	1.24 ± 0.39	29.54 ± 9.82

## Discussion

Major findings emanating from our work are that the remarkable antileishmanial activity exhibited by PNH and (PNE) fractions *in vitro* was interceded via apoptosis and the *in vivo* therapeutic potential was accompanied by Th1 stimulation without any potential toxicity. We have previously reported potent leishmanicidal activity of PNH and PNE against *L. donovani* promastigotes and amastigotes and identified the plant secondary metabolites present in PNH and PNE. We have found that PNH was majorly constituted by *trans*-β-caryophyllene (22.28%), whereas PNE was dominantly constituted by PIP (70.36%) (Chouhan et al., 2014b).

PNH and PNE induced apoptosis in *L. donovani* promastigotes which is the preferred mode of cell death. *L. donovani* promastigotes are known to undergo apoptosis in response to various plant extracts and plant secondary metabolites (Chouhan et al., 2015 and references therein). Induction of apoptosis by PNH and PNE in *L. donovani* promastigotes was evidenced by PS externalization detected by annexin-PI co-staining, DNA fragmentation demonstrated by TUNEL assay and cell cycle analysis, depolarization of MMP/Ψm by JC-1 staining and generation of ROS by H_2_DCFDA staining. H_2_DCFDA is a widely used fluorescent probe for detection of ROS despite the lack of specificity. In addition to ROS, it can also detect other oxidants such as reactive nitrogen species (RNS) in a cell (Koopman et al., 2006), both of which are however known to induce apoptosis (Shen and Liu, 2006). Thus, H_2_DCFDA staining gives a broad idea about oxidant levels in a cell but the non-specificity of the probe makes it difficult to identify the nature of the oxidants (Wardman, 2007). Since, high level of oxidants generate oxidative stress that triggers apoptosis (Yin et al., 2003), it can be concluded that high levels of ROS detected in PNH and PNE treated promastigotes may have aided in apoptosis induction. Pentamidine has been in regular use as a positive control in several antileishmanial studies (dos Santos et al., 2011; Sawadogo et al., 2012; Benitez et al., 2013) including earlier studies performed by us (Chouhan et al., 2014b, 2015; Islamuddin et al., 2014). Since, pentamidine is also known to induce apoptosis in *Leishmania* promastigotes (Singh and Dey, 2007) it was included as positive control for all apoptotic studies. Ethanolic extract of *P. betle* had been shown to induce apoptosis in *L. donovani* promastigotes by disrupting Ψm and causing DNA fragmentation including generation of nicked DNA and cell cycle arrest by Sarkar et al. (2008). Our results also indicated that *P. nigrum* bioactive fractions bore apoptosis inducing potential in *L. donovani* promastigotes as evidenced by Annexin-PI co-staining, TUNEL assay, cell cycle analysis, study of MMP/Ψm and detection of ROS. Trans-β-caryophyllene, a major component of PNH is also known to induce apoptosis in tumor cell lines (Amiel et al., 2012) and may have contributed to the apoptotic potential of PNH. Several authors have used *Leishmania* promastigotes as primary choice for rapid drug screening and also for elucidation of apoptosis related phenomena in place of ‘amastigotes’ which are clinically relevant stage of the parasite (Machado et al., 2012; Sifaoui et al., 2014; Sousa et al., 2014; da Silva et al., 2015). Though, here we have demonstrated apoptosis inducing capacity of our bioactive fractions, a more definitive conclusion can only be obtained by further assessing the apoptosis inducing potential of PNH and PNE in *L. donovani* amastigotes.

*In vivo* antileishmanial and immunomodulatory potential of *P. nigrum* bioactive fractions was appraised *in vivo* in BALB/c mice. BALB/c mice are highly susceptible to *L. donovani* infection (Rolao et al., 2003) and have been proven to be immensely useful for illumination pertaining to pathogenesis and resolution of disease (Mahmoudzadeh-Niknam et al., 2007). PNH and PNE at 200 mg/ kg bw rendered remarkable protection whereas partial recovery from disease was evident at 100 mg/kg bw dose. Despite several antileishmanial studies from numerous plants of the genus *Piper* (Torres-Santos et al., 1999; Hermoso et al., 2003; Bodiwala et al., 2007; Sarkar et al., 2008; Flores et al., 2009; Rodrigues-Silva et al., 2009; Monzote et al., 2010; Cruz et al., 2011; Carrara et al., 2013), there are no reports on *in vivo* antileishmanial potential of *P. nigrum* extracts or pure compounds isolated from *P. nigrum* to the best of our knowledge. However, *in vivo* antileishmanial efficacy of PIP and its different formulations have been earlier reported. Veerareddy et al. (2004) demonstrated low curative efficacy of PIP in comparison to its lipid nanosphere formulation in *L. donovani* infected BALB/c mice. Similarly in hamster model also, free PIP was found to be less effectual than its mannose-coated liposomal formulation in resolving VL infection (Raay et al., 1999). In our studies, we also observed that PIP rendered moderate protection *in vivo* and was less effectual in comparison to PNH and PNE. We have already established superior efficacy of PNH and PNE over PIP against *L. donovani* promastigotes (Chouhan et al., 2014b) and previous *in vitro* studies in this regard have also pointed out that PIP is more efficacious against *L. amazonensis* (new world species) rather than *L. donovani* (Kapil, 1993; Singh et al., 2010; Ferreira et al., 2011). However, it is also likely that moderate antileishmanial efficacy exhibited by PIP may have compounded the leishmanicidal activity of PNH and PNE.

**FIGURE 10:**
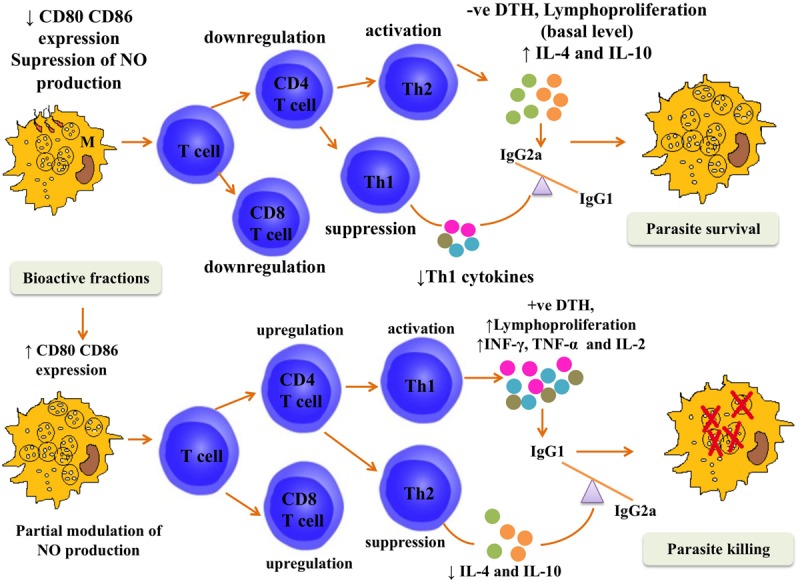
**Postulated mechanism for *in vivo* efficacy of *P. nigrum* bioactive fractions.** The figure depicts that during the course of *L. donovani* infection in BALB/c mice, the immune response was tilted toward Th2 type. The co-stimulatory molecules CD80 and CD86 were downmodulated and NO production was suppressed. CD4^+^ and CD8^+^ T cell numbers also declined and the immune response was driven toward Th2 type under the influence of elevated IL-4 and IL-10 levels leading to parasite survival characterized by negative DTH and only basal levels of lymphoproliferation. This immune containment was reversed following treatment with *P. nigrum* bioactive fractions. The CD80–CD86 expression was elevated, NO was enhanced, and splenic CD4^+^ and CD8^+^ T numbers were also augmented. Enhanced secretion of proinflammatory cytokines (INF-γ, TNF-α, and IL-2) further strengthened the Th1 potentiation as was evident from positive DTH and strong lymphoproliferative response and led to parasite killing.

The therapeutic cure by *P. nigrum* bioactive fractions was steered by Th1 polarization (Summarized in **Figure 10**). *P. nigrum* hosts numerous biological activities including immunomodulatory where it has been shown to exhibit typical Th1 favoring potential including splenocyte lymphoproliferation and upregulation of Th1 cytokines (Majdalawieh and Carr, 2010). In accordance, with these earlier findings, our data also elaborated the potent immunostimulatory potential of *P. nigrum* bioactive fractions that might have augmented their *in vivo* therapeutic potential. AmB used as a positive control for *in vivo* experiments, is known to be immunomodulatory (Reyes et al., 2000) coupled to its antileishmanial activity. In experimental VL, AmB has been demonstrated to induce lymphoproliferation and NO production, elicit DTH, and alter IgG isotype levels in *L. donovani* infected BALB/c mice (Banerjee et al., 2008).

PNH and PNE (200 mg/kg bw) treated mice displayed strong DTH responses which were weaker at 100 mg/kg bw and in PIP treated group. DTH reaction is elicited in response to parasite invasion and aids in elimination of the pathogen (Kaur et al., 2010) as well as in the context of VL it denotes full recovery from infection with lesser risk of relapse (Grimaldi and Tesh, 1993). *Leishmania* parasites paralyze host immunity in more than one ways and depression of T cell proliferative responses is very common during the infection period (Carvalho et al., 1994). Treatment with PNH and PNE (200 mg/kg bw) abrogated the T cell anergy and strong *in vitro* recall responses were evident in response to restimulation of splenocytes with SLA. Lymphoproliferative responses were less pronounced in case of PIP and at 100 mg/kg bw dose of PNH and PNE. Conventionally, CD4^+^ T lymphocytes were considered more crucial in containing leishmaniasis but of late, important participatory role of CD8^+^ T lymphocytes in clearing *Leishmania* infection has been demonstrated (Beattie et al., 2010; Kaushal et al., 2014). *L. donovani* is known to suppress the expansion and effector function of CD4^+^ and CD8^+^ cells during the course of disease (Joshi et al., 2009; Esch et al., 2013). PNH and PNE significantly enhanced CD4^+^ population at 200 mg/kg bw whereas at 100 mg/kg bw the CD4^+^ expression was restored to normal range. Changes in CD8^+^ T cell population varied amongst the different treatment groups. While PNH significantly upregulated the number of CD8^+^ T lymphocytes in comparison to INF group, such changes were less apparent in AmB and PIP treated groups. The elevation in CD8^+^ population after PNE treatment at 200 mg/kg bw was comparatively less than that observed at 100 mg/kg bw. This may be attributed to the fact that pro- or anti-immunopotentiating effects of natural immunomodulators can be dose dependent (Spelman et al., 2006) and has also been evidenced earlier in case of other plant extracts (SaiRam et al., 1997).

Cytokine milieu in VL is muddled and anti-inflammatory or Th2 cytokines dominate the diseased state whereas cure is propelled by secretion of Th1 or pro-inflammatory cytokines. Treatment with PNH elevated INF-γ, TNF-α, and IL-2 levels whereas PNE exhibited variable effect. PNE did not significantly elevate TNF-α and IL-2 but enhanced INF-γ levels. INF-γ is a characteristic pro-inflammatory cytokine, which activates macrophages and also promotes the class switching of B cells to produce more of IgG2a. INF-γ is known to work in synergy with both TNF-α and IL-2 in maintaining a Th1 polarized environment (Sharma et al., 2012), TNF-α is also known to induce macrophage’s NADPH oxidase and aids in macrophage activation (Diaz-Gandarilla et al., 2013). IL-4 and IL-10 levels were also downregulated after bioactive fractions treatment and PNH treatment remarkably declined IL-10 secretion. The stimulation of IgG2a and a concomitant decrease in IgG1 levels after bioactive fractions treatment can be well correlated with enhanced INF-γ production and suppression of IL-4 secretion by PNH and PNE. IL-10 has been designated as master puppeteer in VL exacerbation. Though, in murine and human VL, INF-γ is produced in sufficient amounts, IL-10 is known to override Th1 stimulatory capabilities of INF-γ by suppressing its production, macrophage activation, downmodulating CD80 and CD86 expression and impairing other accessory cell functions (Couper et al., 2008).

The interaction between T cell and major histocompatibility complex is cemented by co-stimulatory molecules including CD80 and CD86. CD80–CD86 expression that was downregulated during infection was revived by treatment with PNH and PNE as well as PIP reflecting that bioactive fractions treatment enhances the ability of macrophages to present leishmanial antigens to T cells. Same has been observed earlier in case of treatment with miltefosine and pyrazinamide (Mendez et al., 2009; Mukhopadhyay et al., 2011).

Nitric oxide induction is another parameter, which indicates Th1 potentiation as it is produced by NOSII activated by Th1 cytokines. *P. nigrum* bioactive fractions along with PIP did not significantly enhance the NO production. Although, there is no dearth of evidence depicting crucial role of NO in killing intracellular *Leishmania* parasites but there are multifarious reports indicating that INF-γ dependent NOSII or NO independent leishmanicidal pathways also exist (Bogdan, 2007 and references therein). This may also be true in case of parasite death induced by *P. nigrum* bioactive fractions. Further, trans-β-caryophyllene (major component in PNH) has been reported to be active against *L. amazonensis* amastigotes in an NO-independent manner (Soares et al., 2013). PIP also failed to induce NO production and similar observation has been reported by others. PIP though inhibited the growth of *L. amazonensis* amastigotes but failed to induce NO production (Ferreira et al., 2011). PIP is also reported to inhibit NO production *in vivo* (Pradeep and Kuttan, 2003) and it also inhibited the growth of *Trypanosoma cruzi* epimastigotes without NO induction (Freire-de-Lima et al., 2008).

*In vivo* toxicity of the bioactive fractions was assessed at higher dose (200 mg/ kg bw) in *L. donovani* infected mice. *In vitro* toxicity of *P. nigrum* bioactive fractions had earlier been studied against RAW macrophages where it was observed that both PNH and PNE possessed high CC_50_ (50% inhibitory concentration against macrophages) of 379.7 and 200 μg ml^-1^, respectively, and were specific against *L. donovani* amastigotes (Chouhan et al., 2014b). Hepatomegaly is one of the characteristic clinical symptoms of VL and during the establishment of infection, renal functions are also severely impaired (Prianti et al., 2007). There was a significant increase in enzymatic activity of SGOT, SGPT, and ALP as well as urea and creatinine concentration in INF and VC groups. Similar observations in case of experimental VL have been demonstrated by others (Pal et al., 2002; Kaur et al., 2013; Joshi et al., 2014). None of the treatment groups significantly altered serum levels of hepatic and renal enzymes. Hepatoprotective potential of *P. nigrum* (Nirwane and Bapat, 2012) and PIP (Sabina et al., 2010) has been earlier reported and our findings also pointed toward inertness of PNH and PNE toward hepatic and renal toxicity. The adversity of antileishmanial chemotherapeutics and even of promising drugs in experimental stage has always been the focal point of criticism. Over the years, the search for potent as well as equally safe or less toxic antileishmanial drugs has been elusive. *P. nigrum* bioactive fractions in addition to being antileishmanial and immunomodulatory, proved to be devoid of hepato- and nephro-toxicity thereby suggesting that these fractions can be a source of safe lead compounds with profound leishmanicidal efficacy.

In summary, drugs with immunomodulatory capacity have evolved as promising therapeutic agents for the cure of infectious diseases. In the present scenario, when conventional therapeutic modalities against VL are of limited efficacy with an escalating threat of resistance, therapeutic switching to drugs which can directly inhibit the growth of *Leishmania* parasites and can liberate the host immune system of parasite induced suppressive effects, is urgently desired. Plants have been a store house of biologically active substances endowed with miscellaneous therapeutic properties and research over decades has demonstrated that plants can yield diverse antileishmanial molecules with immunomodulatory properties. Present study clearly illustrated the promising leishmanicidal efficacy of *P. nigrum* bioactive fractions, which was substantiated by its Th1 stimulatory potential with no significant toxicity on host liver and kidney. Thus, our findings point toward the candidacy of *P. nigrum* as a source of potent and safe antileishmanial compounds which after careful evaluation may benefit VL therapy as adjunct modality to complement the existing drugs.

## Author Contributions

GC, MI: designed and conceived the study, performed experiments, analyzed, and interpreted the data. MW: aided in carrying out *in vivo* experiments. DS, HO, and HH: contributed to reviewing of manuscript. FA: conceived and supervised the work, and contributed to designing of experimental plan and execution of work along with analysis and interpretation of data, manuscript writing and reviewing the manuscript. All the authors approved final version of the research article.

## Conflict of Interest Statement

The authors declare that the research was conducted in the absence of any commercial or financial relationships that could be construed as a potential conflict of interest.

## Abbreviations

AIDS: acquired immunodeficiency syndrome
ALP: alkaline phosphatase
AmB: Amphotericin B
ANOVA: one-way analysis of variance
APC: allophycocyanin
BSA: bovine serum albumin
CFSE: carboxyfluorescein succinimidyl ester
CL: cutaneous leishmaniasis
CMI: cell-mediated immunity
ConA: Concanavalin A
CPCSEA: Committee for the purpose of control and supervision of experiments on animals
DMSO: dimethylsulfoxide
DTH: delayed type hypersensitivity
FBS: fetal bovine serum
FITC: fluorescein isothiocyanate
FT: freeze thawed antigen
GC-MS: Gas chromatography-Mass spectrometry
HEPES: 4-(2-hydroxyethyl)-1-piperazine-ethanesulfonic acid
H_2_DCFDA: 2′, 7′- dichlorodihydrofluorescein-diacetate
IgG: Immunoglubulin G
i.p.: intraperitoneal
i.v.: intravenous
INF: infection control
JC-1: 5,5′,6,6′-tetrachloro-1,1′,3,3′-tetraethylimidacarbocyanine iodide
JHAEC: Jamia Hamdard Animal Ethics Committee
LDU: leishman donovan units
MFI: mean fluorescence intensity
MMP/Ψm: mitochondrial membrane potential
NO: nitirc oxide
NOSII: nitric oxide synthase II
PBS: phosphate buffered saline
PE: Phycoerythrin
PI: propidium iodide
PIP: piperine
PNE: *P. nigrum* seeds ethanolic fraction
PNH: *P. nigrum* seeds hexane fraction
PS: phosphatidylserine
RBCs: red blood cells
ROS: reactive oxygen species
RPMI: Roswell Park Memorial Institute
RT: room temperature
SEM: standard error of mean
SGOT: serum glutamate oxaloacetate transaminase
SGPT: serum glutamate pyruvate transaminase
SLA: soluble leishmanial antigen
Th: T helper type
TUNEL: terminal deoxynucleotidyltransferase (TdT) mediated dUTP (deoxyuridine triphosphate) nick end labeling
VC: vehicle control
VL: visceral leishmaniasis
