# Corrigendum: Leishmanicidal Activity of *Piper nigrum* Bioactive Fractions is Interceded via Apoptosis *In Vitro* and Substantiated by Th1 Immunostimulatory Potential *In Vivo*

**DOI:** 10.3389/fmicb.2016.00944

**Published:** 2016-06-20

**Authors:** Garima Chouhan, Mohammad Islamuddin, Muzamil Y. Want, Hani A. Ozbak, Hassan A. Hemeg, Dinkar Sahal, Farhat Afrin

**Affiliations:** ^1^Parasite Immunology Laboratory, Department of Biotechnology, Faculty of Science, Jamia Hamdard (Hamdard University)New Delhi, India; ^2^Department of Medical Laboratories Technology, Faculty of Applied Medical Sciences, Taibah UniversityMedina, Saudi Arabia; ^3^Malaria Research Group, International Centre for Genetic Engineering and BiotechnologyNew Delhi, India

**Keywords:** visceral leishmaniasis, antileishmanial, *Piper nigrum*, immunomodulatory, leishmanicidal, *Leishmania donovani*, apoptosis

Reason for Corrigendum:

Due to an oversight, there was a mistake in the affiliation of the Corresponding Author Farhat Afrin, as published. Also, in the original article, we have neglected to thank our sponsors Deanship of Scientific Research, Taibah University, Medina, Saudi Arabia, grant number 7127 to FA and HH. The correct Acknowledgments should have been published as:

## Acknowledgments

Present study was financially aided by Department of Science and Technology, Department of Biotechnology and Central Council for Research in Unani Medicine (CCRUM), Government of India. GC was formerly recipient of Senior Research Fellowship from CCRUM and Basic Research Fellowship from University Grants Commission (UGC), under UGC-Special Assistance Grant (SAP) received by Department of Biotechnology, Jamia Hamdard, during the period of study. We profoundly thank Dr. N. Ali, Indian Institute of Chemical Biology, Kolkata, India, for providing us L. *donovani* culture. Our thanks are due to Mr. T. A. Nagarjuna and Dr. V. Gupta, BD-JHFACS Academy, Jamia Hamdard, for their immense help and cooperation in flow cytometry work. We are also thankful to Mrs. F. Ahmad who extended help in plant collection and extraction. Our collaborative Microbiology Research Group at Faculty of Applied Medical Sciences, Taibah University, Kingdom of Saudi Arabia is gratefully acknowledged for meeting the publication cost and process apart from their significant roles and scientific participation in the work. The present study was supported by theDeanship of Scientific Research (grant number 7127), Taibah University, Medina, Saudi Arabia, Department of Science and Technology, Department of Biotechnology and Central Council for Research in Unani Medicine (CCRUM), Government of India.

The authors apologize for this oversight. This error does not change the scientific conclusions of the article in any way.

## Author contributions

GC, MI: Designed and conceived the study, performed experiments, analyzed, and interpreted the data. MW: aided in carrying out in vivo experiments. DS, HO, and HH: contributed to reviewing of manuscript. FA: conceived and supervised the work, and contributed to designing of experimental plan and execution of work along with analysis and interpretation of data, manuscript writing and reviewing the manuscript. All the authors approved final version of the research article.

### Conflict of interest statement

The authors declare that the research was conducted in the absence of any commercial or financial relationships that could be construed as a potential conflict of interest.

